# Mitigation of heterocyclic amines, polycyclic aromatic hydrocarbons, and acrylamide in air-fried chicken and beef: effects of cooking methods and marinades

**DOI:** 10.1007/s10068-025-02005-8

**Published:** 2025-09-27

**Authors:** Jungwon Kwon, Inhwan Kim, Kwang-Won Lee, MunYhung Jung, Hyunjun Lee, Seoyeong Kim, BoKyung Moon, Jihyun Lee

**Affiliations:** 1 Department of Food Science and Technology, Chung-Ang University, Anseong, 17546 Republic of Korea; 2 Department of Biotechnology, College of Life Science and Biotechnology, Korea University, Seoul, 02841 Republic of Korea; 3 Department of Food Science and Biotechnology, Graduate School, Woosuk University, Wanju-gun, Jeonbuk 55338 Republic of Korea; 4 Department of Food and Nutrition, Chung-Ang University, Anseong, 17546 Republic of Korea; 5 Department of Food and Nutrition, College of Human Ecology, Seoul National University, Seoul, 08826 Republic of Korea

**Keywords:** PhIP, AαC, Milk, Beer, Turmeric

## Abstract

This study evaluated how air frying conditions, temperature, time, searing, marination, and spices, affect the formation of heterocyclic amines (HCAs), polycyclic aromatic hydrocarbons (PAHs), and acrylamide in whole chicken and beef steak. 10 HCAs, 4 PAHs, and acrylamide were quantified using UHPLC-(ESI)-QqQ and GC–MS. Higher temperatures and searing increased HCAs and acrylamide levels, especially in beef. In chicken, marination with milk or beer has reduced HCAs by up to 60.6%, with no significant effect on PAHs or acrylamide. In beef, turmeric, rosemary, and garlic suppressed HCA formation, with turmeric showing the greatest reducing effect (69.4%). PAHs were only found in chicken, with HCAs and PAHs showing a strong correlation (*p* < 0.01), suggesting similar formation pathways. These findings highlight the role of specific cooking methods and marination ingredients, especially turmeric, in reducing harmful compounds in air-fried meats, offering guidance for safer home and industrial cooking practices.

**Supplementary Information:**

The online version contains supplementary material available at 10.1007/s10068-025-02005-8.

## Introduction

During high-temperature cooking, various harmful compounds, including heterocyclic amines (HCAs), polycyclic aromatic hydrocarbons (PAHs), and acrylamide are formed mainly through the Maillard reaction and pyrolysis of food components. HCAs are generated when creatine or creatinine, amino acids, and sugars react at 150–250 °C, producing thermic HCAs (Jägerstad et al., 1998), while pyrolytic HCAs are formed above 250 °C via amino acid degradation (Matsumoto et al., 1981). PAHs, consisting of multiple aromatic rings, are produced by the pyrolysis of organic compounds such as fat, carbohydrates, and protein above 200 °C (Adeyeye, 2020). Acrylamide is mainly formed via the Maillard reaction between asparagine and reducing sugars, particularly during high-temperature processes like frying, roasting, or baking (Kumar et al., 2018).

Numerous HCAs such as 2-amino-3-methyl-3H-imidazo[4,5-f]quinoline (IQ), 2-amino-9H-pyrido[2,3-b]indole (AαC), 2-amino-3-methyl-9H-pyrido[2,3-b]indole (MeAαC), 2-amino-3,8-dimethylimidazo[4,5-f]quinoxaline (MeIQx), 2-amino-3,4-dimethyl-3H-imidazo[4,5-f]quinoline (MeIQ), 2-amino-1-methyl-6-phenylimidazo[4,5-b]pyridine (PhIP), 3-Amino-1,4-dimethyl-5H-pyrido[4,3-b]indole (Trp-P-1), and 3-Amino-1-methyl-5H-pyrido[4,3-b]indole (Trp-P-2) have been identified in cooked meats, with many classified by the International Agency for Research on Cancer (IARC) as Group 2A (probable carcinogens) or Group 2B (possible carcinogens) (IARC, 2025). HCAs have been associated with increased risks of cancers, including colorectal, prostate, mammary, and liver cancers (Smolińska and Paluszkiewicz, 2010). PAHs such as benzo[a]pyrene (B[a]P), benzo[b]fluoranthene (B[b]F), benzo[a]anthracene (B[a]A), and chrysene (CRY) are mutagenic and genotoxic, with B[a]P classified as a Group 1 and B[b]F, B[a]A, and CRY classified as a Group 2B by IARC (EFSA, 2008; IARC, 2025). These compounds can interfere with DNA replication and cell division, contributing to carcinogenesis (da Silva Junior et al., 2021). Acrylamide, also a probable human carcinogen (Group 2A), has been linked to several types of tumors and neurotoxicity in animal studies (Zamani et al., 2017).

Global meat consumption is increasing rapidly, projected to rise from 32 to 52 kg per capita by 2050 (Gurría, 2017). In Korea, meat consumption—particularly of chicken and beef—has expanded significantly. This has raised concerns about the formation of heat-induced toxic compounds during home and commercial cooking. HCAs have been detected in barbecued beef steak at concentrations up to 13.52 ng/g (Oz and Yuzer, 2016), and PAHs in grilled beef have been found at levels between 3.1–43.7 ng/g depending on cooking temperature (Ahmad Kamal et al., 2018). Acrylamide formation is also affected by cooking methods, with the highest levels observed in microwaved and roasted meat products (Michalak et al., 2017; Michalak et al., 2017).

To reduce these compounds, strategies such as the use of natural antioxidants and marination have been proposed. Antioxidants from spices (e.g., garlic, pepper, onion) or plant-based ingredients can scavenge free radicals and suppress the Maillard reaction intermediates that lead to HCAs and PAHs formation. Additionally, marinades—especially those based on beer or red wine—have been shown to reduce HCAs and PAHs by limiting direct heat exposure (Melo et al., 2008). However, certain antioxidant-rich additives may also enhance acrylamide formation under specific cooking conditions (Zeng et al., 2017).

Recently, air fryers have gained popularity due to their convenience and perceived health benefits. Air frying utilizes circulating hot air and fine oil droplets to cook food more evenly and with less fat. These cooking characteristics may contribute to the reduced formation of harmful compounds such as HCAs, PAHs, and acrylamide. For instance, Lee et al. (2020) reported that air-fried chicken wings, thighs, and breasts contained lower levels of PAHs and acrylamide than the corresponding deep-fried samples. However, this comparison was based on a single fixed cooking temperature and time, which may not adequately represent the variability of real-world cooking practices. In addition to cooking methods, the use of spices has also been associated with reduced HCA formation. In a previous study (Kwon et al., 2023), both air frying and spice addition were shown to significantly inhibit HCA formation in chicken wings and pork belly. Nevertheless, that analysis was limited to HCAs, without consideration of other heat-induced toxicants such as PAHs or acrylamide. HCAs, PAHs, and acrylamide share common formation pathways, as they are produced through Maillard reactions between amino acids and reducing sugars under high-temperature, prolonged cooking conditions (Adeyeye, 2020; Zhang et al., 2009). Therefore, a comprehensive evaluation of cooking conditions on the simultaneous formation of these toxic compounds is required, although integrated analyses of this type remain limited to date. One example is the study by Yoon et al. (2024), which quantified HCAs, PAHs, and acrylamide in commonly consumed foods prepared using an air fryer and conducted a dietary exposure assessment.

Given that antioxidant interventions may differentially influence the formation of these three toxicants, and considering the increasing popularity of air frying, additional comprehensive data are required. In particular, the combined effects of cooking parameters (e.g., temperature, time), marination, and spice addition on the formation of HCAs, PAHs, and acrylamide in air-fried meat remain insufficiently characterized. To address this gap, common air fryer recipes for chicken and beef were reviewed from cookbooks and online sources to establish representative cooking conditions. For chicken, marination prior to cooking was frequently observed; therefore, beer and milk were selected as representative marinades based on their distinct composition and traditional culinary use. Beer contains polyphenols and other Maillard reaction-modulating compounds derived from hops and malt (Piazzon et al., 2010), which have been reported to reduce HCA and PAH formation. In contrast, milk contains proteins and reducing sugars that may interact differently with heat-induced reactions (Barbanti and Pasquini, 2005), potentially influencing the formation of acrylamide and HCAs. For beef, rosemary, turmeric, and garlic were chosen as representative spices due to their frequent use in culinary practice. These spices provide diverse antioxidant constituents, including phenolic compounds and curcuminoids (Afonso et al., 2013; Beato et al., 2011; Kocaadam and Şanlier, 2017), which may modulate the generation of HCAs, PAHs, and acrylamide during cooking. Accordingly, this study aimed to systematically evaluate the effects of air frying parameters—including temperature, time, marination type, and spice addition—on the formation of HCAs, PAHs, and acrylamide in chicken and beef, with the goal of providing mechanistic insights and practical recommendations for mitigating toxicant formation in air-fried meats.

## Materials and methods

### Chemicals and reagents

10 HCAs (i.e., AαC, MeAαC, IQ, MeIQx, MeIQ, PhIP, Trp-P-1 acetate, Trp-P-2 acetate, Harman, and Norharman) and internal standards for HCAs (i.e., MeAαC-*d*_3_, IQ-*d*_3_, MeIQx-*d*_3_, MeIQ-*d*_3_, Trp-P-2-^13^C_2_-^15^N acetate, PhIP-*d*_3_, Harman-*d*_3_, and Norharman-*d*_7_) were obtained from Cayman Chemical (Ann Arbor, MI, USA), Sigma Aldrich (St. Louis, MO, USA), or Toronto Research Chemical (Toronto, Ontario, Canada). All authentic standards of HCAs and internal standards had purities of at least 95%. Acrylamide and ^13^C_3_-acrylamide were obtained from Sigma Aldrich and Cambridge Isotope Laboratories, Inc. (Andover, MA, USA), respectively. The PAH standard solution EPA 525 PAH Mix A, containing B[*a*]A (99%), B[*a*]P (96%), B[*b*]F (98%), and CRY (98%), was acquired from Sigma Aldrich. Internal standards for PAHs including B[*a*]A-*d*_12_ (98%), B[*a*]P-*d*_12_ (98%), B[*b*]F-*d*_12_ (98%), and CRY-*d*_12_ (98%) were purchased from Sigma Aldrich. Sodium hydroxide, ammonium formate, dichloromethane (DCM), and ethyl acetate were purchased from Sigma-Aldrich. Acetonitrile (ACN) and methanol (MeOH) were purchased from J.T. Baker (Radnor, PA, USA) and Honeywell (Charlotte, NC, USA), respectively.

### Meat samples

**Fig. 1:**
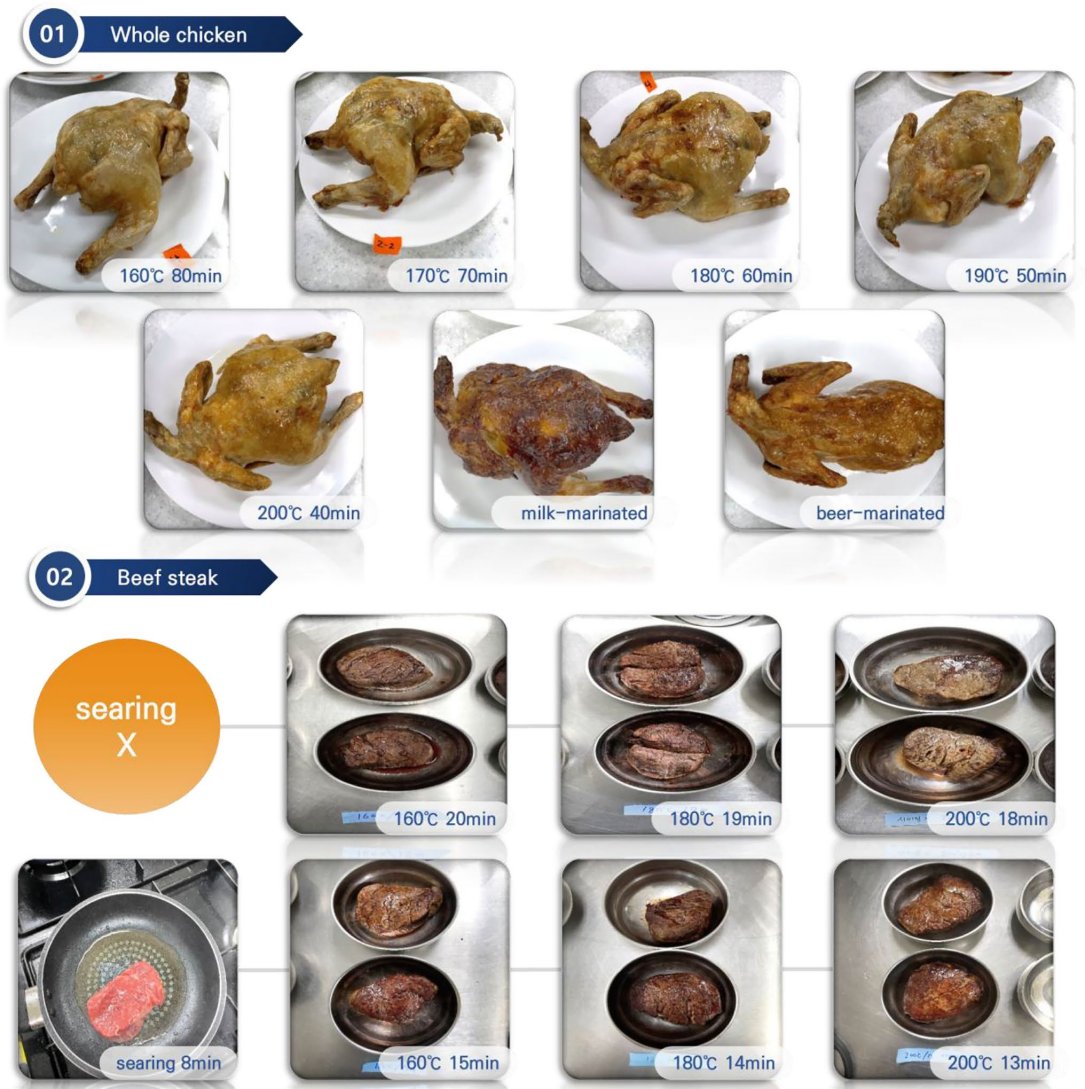
Pictures of whole chicken and beef steak after air frying under various conditions

The whole chicken and beef tenderloin were obtained from local markets located in Anseong, Korea. The samples were cooked using an air fryer (Fig. 1). Cooking recipes published by the equipment makers were used for cooking whole chicken and beef tenderloin. Whole chicken was cooked until the internal temperature reached 75 °C (USDA Food Safety and Inspection Service, 2015), and beef tenderloin was cooked to 72 °C, corresponding to a well-done state (Torun et al., 2023). The whole chicken without marination was air-fried at 160 °C for 80 min, 170 °C for 70 min, 180 °C for 60 min, 190 °C for 50 min, and 200 °C for 40 min. For the marinade treatment group, whole chicken samples were marinated for 30 min at 4 °C, with 500 mL of milk or beer. The whole chicken samples were turned occasionally (every 15 min) to ensure even marination. The samples were taken from the marinade and lightly dried before being air-fried at 200 °C for 40 min. About 180–200 g of raw beef was used to cook beef steak. The beef steak was divided into two groups: air-fried beef steak without searing and air-fried beef steak with searing. The air-fried beef steak without searing was cooked at 160 °C for 20 min, 180 °C for 19 min, or 200 °C for 18 min. The air-fried beef steak with searing was cooked at 160 °C for 15 min, 180 °C for 14 min, or 200 °C for 13 min. Before air frying, each side of the beef tenderloin was seared by pan-frying at 180 ℃ for 4 min. Raw meat was set as a control. Additionally, beef tenderloin (per 100 g of meat) was seasoned with rosemary, turmeric, and garlic (0.5 g per 100 g of meat) before air-frying for 18 min at 200 °C.

All samples were cooked in triplicate using three air fryers. All cooked samples were homogenized together to make a composite sample. In the case of whole chicken samples, bones were removed and only the edible parts (meat) were homogenized. This compositing approach was chosen to reflect typical whole-chicken consumption patterns rather than focusing on a specific cut. Before analysis, the samples were stored at -80 °C.

### Analysis of HCAs in meat samples by UHPLC-(ESI)-QqQ

HCAs extraction was performed following the methods used in our previous study (Kwon et al., 2023; MFDS, 2017). HCAs extraction was performed with sodium hydroxide aqueous solution and ACN. The extract was eluted through Chem Elut cartridge (Agilent Technologies) and Oasis HLB SPE cartridge (Waters, Milford, MA, USA). Then, HCAs content was analyzed by using UHPLC-(ESI)-QqQ (Agilent 1290 infinity UHPLC coupled to Agilent 6470 triple quadrupole mass spectrometer; Agilent Technologies). The HCAs in meat samples were separated on an Acquity BEH C18 column (2.1 × 100 mm, 1.7 μm; Waters). The mobile phases were 30 mM ammonium formate (A) and MeOH (B). The multiple reaction monitoring (MRM) mode was used for determination of HCAs, as shown in Table S1 (MFDS, 2017).

### Analysis of PAHs in meat samples by GC–MS

Extraction of PAHs were determined as described previously (Kim et al., 2021). Briefly, a reflux extractor was used to extract PAHs from fat tissues of food through liquid–liquid extraction after alkaline decomposition after adding 1 mL of the internal standard (consisting of 100 µg/L B[*a*]A-*d*_12_, B[*b*]F-*d*_12_, B[*a*]P-*d*_12_, and CRY-*d*_12_) to the meat sample (5 g). The extract was concentrated with a rotary evaporator (N-1110; EYELA) and filtered through Sep-Pak silica cartridges (Waters) with 5 mL of hexane and 15 mL of hexane-DCM (3:1, v/v). The filtrate was concentrated under N_2_ gas at 40 °C. The concentrate was redissolved with 1 mL of DCM and filtered. The PAHs were analyzed using a model 7890B gas chromatograph (Agilent Technologies) equipped with an Zebron ZB-PAH-SeleCT column (40 m × 0.18 mm, 0.14 μm; Phenomenex, Torrance, CA, USA). The analysis method including carrier gas, flow rate, injection volume, and column oven temperature was conducted as our previous study (Kim et al., 2021). The PAHs contents in the samples extracts were determined by selected ion monitoring (SIM) mode. The selected target ions (m/z) for PAH quantitation were as follows: B[*a*]A (228), B[*b*]F (252), B[*a*]P (252), CRY (228). Selected ion fragments for the identification of analytes were as follows: B[*a*]A (226, 229), B[*b*]F (250, 253), B[*a*]P (250, 253), and CRY (226, 229). The limit of detection and quantitation for PAHs analysis ranged from 0.038 to 0.185 μg/kg and from 0.114 to 0.560 μg/kg, respectively, with recovery values between 87.64% and 112.25%. Detailed validation procedures and additional parameters are described in our previous study (Kim et al., 2021).

### Determination of acrylamide by UHPLC-(ESI)-QqQ

To extract acrylamide, the homogenized meat sample (1 g) was weighed. Subsequently, 9 mL of water and 1 mL of ^13^C_3_-acrylamide (an internal standard, 2 µg/mL) were added. A shaker (Mini-G; Elim Global, Staten Island, NY, USA) was used to shake it for 20 min at 250 rpm. The extracts were centrifuged at 3,500 rpm for 5 min (Combi 408; Hanil, Daejeon, Korea), and 5 mL of the supernatant was filtered. Next, 1.5 mL of the supernatant was introduced onto a pre-conditioned Strata SPE cartridge. Subsequently, water (1.5 mL) was added into the Strata-X SPE cartridge, and the eluent was collected. From the Strata SPE, 0.5 mL of eluent was passed through a pre-conditioned Bond Elut AccuCAT SPE cartridge, and the eluent was discarded. The remaining 1.0 mL of eluent obtained from the first SPE cartridge was introduced to the Bond Elut AccuCAT SPE, and the eluent was collected.

The collected eluent was analyzed using UHPLC-(ESI)-QqQ (Nexera X2 UHPLC coupled to TQ 8040; Shimadzu, Kyoto, Japan). Acrylamide in the extracts was determined using a Phenomenex Polar C18 column (2.1 mm × 150 mm, 2.6 µm; Phenomenex) maintained at 26 °C. The mobile phase was an isocratic mixture of 0.2% acetic acid and 5% methanol. The flow rate was 0.2 mL/min and ESI positive mode was employed. MRM transitions were utilized for the quantitation of acrylamide and ^13^C_3_-acrylamide, which were m/z 72 > 55 and m/z 75 > 58, respectively. For the identification of acrylamide and ^13^C_3_-acrylamide, selected ion fragments were m/z 72 > 27 and 75 > 29, respectively.

### Statistical analysis

IBM SPSS Statistics V23 (SPSS, Inc., Chicago, IL, USA) was used for statistical analysis. Significant differences in HCAs, PAHs, and acrylamide levels among the samples were confirmed by an analysis of variance (ANOVA). The post-hoc test was conducted by Duncan’s tests (*p* < 0.05). The relationships between contents of HCAs, PAHs, and acrylamide were analyzed by Spearman correlation coefficients.

## Results and discussion

### Effects of air frying conditions on HCAs formation in whole chicken and beef steak

The effects of cooking conditions on HCAs formation were evaluated using air-fried whole chicken and beef steak. Tables 1 and 2 present the contents of HCAs detected at various air-frying temperatures and times. Among the detected HCAs, Harman and Norharman are not classified as carcinogenic; thus, comparisons of cooking conditions focused on the total content of the other 8 carcinogenic HCAs.

**Table 1:** Effects of different cooking conditions on the production of HCAs, PAHs, and acrylamide (μg/kg) in whole chicken using an air fryer

Cooking temperature and time	AαC	MeAαC	IQ	MeIQx	MeIQ	PhIP	Trp-P-1	Trp-P-2	Harman	Norharman	Total 8 HCAs*	B[*a*]A	B[*b*]F	B[*a*]P	CRY	Total PAHs	Acrylamide
Control (raw chicken)	0.13 ± 0.02^ab^	0.14 ± 0.01^a^	N.D	N.D	N.D	0.21 ± 0.04^e^	N.D	N.D	0.33 ± 0.05 ^d^	3.23 ± 0.53^a^	0.49 ± 0.02^d^	N.D	N.D	0.028 ± 0.05^a^	N.D	0.028 ± 0.05^a^	1.09 ± 0.46^a^
160 °C, 80 min	0.14 ± 0.02^ab^	0.13 ± 0.01^a^	N.D	N.D	N.D	0.76 ± 0.14^d^	N.D	N.D	0.70 ± 0.10 ^b^	2.75 ± 0.37^ab^	1.03 ± 0.11^c^	N.D	N.D	0.032 ± 0.05^a^	N.D	0.032 ± 0.05^a^	2.15 ± 1.05^a^
170 °C, 70 min	0.15 ± 0.02^ab^	0.14 ± 0.01^a^	N.D	N.D	N.D	1.04 ± 0.12^c^	N.D	N.D	0.38 ± 0.02^d^	1.50 ± 0.25^c^	1.33 ± 0.11^bc^	N.D	N.D	0.069 ± 0.06^a^	N.D	0.069 ± 0.06^a^	1.31 ± 0.49^a^
180 °C, 60 min	0.17 ± 0.03^a^	0.15 ± 0.02^a^	N.D	N.D	N.D	1.18 ± 0.19^bc^	N.D	N.D	0.81 ± 0.02^a^	2.56 ± 0.33^b^	1.51 ± 0.18^b^	N.D	N.D	0.077 ± 0.07^a^	N.D	0.077 ± 0.07^a^	1.15 ± 0.95^a^
190 °C, 50 min	0.12 ± 0.02^b^	0.13 ± 0.00^a^	N.D	N.D	N.D	1.37 ± 0.18^b^	N.D	N.D	0.59 ± 0.05^c^	1.71 ± 0.22^c^	1.62 ± 0.18^b^	N.D	N.D	1.141 ± 0.03^a^	N.D	1.141 ± 0.03^a^	2.06 ± 1.38^a^
200 °C, 40 min	0.17 ± 0.03^ab^	0.15 ± 0.01^a^	N.D	0.08 ± 0.01^a^	N.D	2.16 ± 0.28^a^	N.D	N.D	0.72 ± 0.02^ab^	1.62 ± 0.23^d^	2.56 ± 0.30^a^	N.D	N.D	0.100 ± 0.10^a^	N.D	0.100 ± 0.10^a^	1.47 ± 1.52^a^

*8 HCAs: AαC, MeAαC, IQ, MeIQx, MeIQ, PhIP, Trp-P-1, and Trp-P-2

N.D. indicates not detected. Data are expressed as mean ± standard deviation

Mean values followed by different lowercase letters within each column for different cooking conditions are significantly different for whole chicken (*p* < 0.05)

**Table 2:** Effects of different cooking conditions on the production of 10 HCAs, 4 PAHs, and acrylamide (μg/kg) in beef steak using an air fryer

Cooking condition	Cooking temperature and time	AαC	MeAαC	IQ	MeIQx	MeIQ	PhIP	Trp-P-1	Trp-P-2	Harman	Norharman	Total 8 HCAs*	B[*a*]A	B[*b*]F	B[*a*]P	CRY	Total PAHs	Acrylamide
-	Control (raw beef)	0.14 ± 0.03^ab^	0.10 ± 0.01^a^	N.D	N.D	N.D	0.40 ± 0.07^d^	N.D	N.D	0.26 ± 0.05^c^	4.18 ± 0.04^a^	0.64 ± 0.05^d^	N.D	N.D	N.D	N.D	N.D	N.D
Air frying only	160 °C, 20 min	0.10 ± 0.02^bcd^	0.10 ± 0.00^ab^	N.D	N.D	N.D	0.58 ± 0.10^ cd^	N.D	N.D	0.41 ± 0.07^bc^	4.30 ± 0.19^a^	0.78 ± 0.09^ cd^	N.D	N.D	N.D	N.D	N.D	N.D
	180 °C, 19 min	0.15 ± 0.02^a^	0.10 ± 0.01^ab^	N.D	N.D	N.D	0.56 ± 0.08^ cd^	N.D	N.D	0.30 ± 0.04^bc^	4.16 ± 0.01^a^	0.81 ± 0.10^ cd^	N.D	N.D	N.D	N.D	N.D	0.39 ± 0.09^d^
	200 °C, 18 min	0.13 ± 0.02^abc^	0.08 ± 0.00^b^	N.D	N.D	N.D	0.63 ± 0.10^bcd^	N.D	N.D	0.43 ± 0.03^bc^	4.15 ± 0.02^a^	0.84 ± 0.08^ cd^	N.D	N.D	N.D	N.D	N.D	0.98 ± 0.08^c^
Searing-Air frying	160 °C, 15 min	0.08 ± 0.01^d^	0.11 ± 0.00^a^	N.D	N.D	N.D	0.84 ± 0.11^bc^	N.D	N.D	0.91 ± 0.16^a^	4.15 ± 0.04^a^	1.03 ± 0.10^bc^	N.D	N.D	N.D	N.D	N.D	N.D
	180 °C, 14 min	0.14 ± 0.02^ab^	0.10 ± 0.02^a^	N.D	N.D	N.D	0.92 ± 0.18^b^	N.D	N.D	0.47 ± 0.06^b^	4.18 ± 0.04^a^	1.15 ± 0.20^b^	N.D	N.D	N.D	N.D	N.D	2.41 ± 0.09^a^
	200 °C, 13 min	0.09 ± 0.02^ cd^	0.10 ± 0.00^ab^	N.D	N.D	N.D	1.92 ± 0.31^a^	N.D	0.09 ± 0.01^a^	1.01 ± 0.17^a^	4.17 ± 0.04^a^	2.19 ± 0.32^a^	N.D	N.D	N.D	N.D	N.D	2.13 ± 0.15^b^

*8 HCAs: AαC, MeAαC, IQ, MeIQx, MeIQ, PhIP, Trp-P-1, and Trp-P-2

N.D. indicates not detected. Data are expressed as mean ± standard deviation

Mean values followed by different lowercase letters within each column for different cooking conditions are significantly different for beef steak (*p* < 0.05)

The results showed that as the air frying temperature increased, the total content of 8 HCAs also increased significantly, due to enhanced dehydration, accelerated conversion of creatine (a precursor of several HCAs), and an intensified Maillard reaction. For instance, the total 8 HCAs content in whole chicken air-fried at 200 °C for 40 min (2.56 μg/kg) was approximately 2.5 times higher than that at 160 °C for 80 min (1.03 μg/kg, *p* < 0.05). The specific HCA profiles also varied with temperature. In whole chicken, AαC, MeAαC, PhIP, Harman, and Norharman were commonly detected, while MeIQx appeared only at higher cooking temperatures. These results contrast with our previous results from air-frying chicken wings, where MeIQx was detected at cooking temperatures ranging from 140 °C to 200 °C (Kwon et al., 2023). This discrepancy may be attributed to differences in fat content between chicken parts. Compared to wings and drumsticks, which are relatively high in fat, whole chicken contains breast meat. Therefore, the fat content of the sample or the HCAs content from cuts such as wings or drumsticks would have been diluted. This trend was consistent regardless of cooking time: lower air-frying temperatures consistently resulted in reduced HCAs levels. Specifically, the lowest HCAs concentration was observed at 140 °C in whole chicken and at 160 °C in beef steak. The increase in HCAs at higher temperatures is consistent with previous reports, which explain that reduced moisture and enhanced conversion of creatinine promote the formation of various imidazoquinoline (i.e. IQ and MeIQ) and imidazoquinoxaline (i.e. MeIQx) compounds (Gibis, 2016). Elevated temperatures accelerate the reaction rates involved in HCAs formation, particularly during grilling.

Searing prior to air frying significantly affected HCAs level in beef steak. When seared beef steak was air-fried at 200 °C for 13 min, the total HCAs content (2.19 μg/kg) was 2.6 times higher than that in non-seared beef steak cooked at the same temperature for 18 min (0.84 μg/kg). PhIP and Harman levels were notably elevated by searing, with PhIP reaching 1.92 μg/kg compared to 0.63 μg/kg in non-seared samples (*p* < 0.05). Trp-P-2 was detected only in the seared sample. However, searing had no significant effect on Norharman levels (*p* > 0.05).

The increase in HCAs due to searing may be attributed to the intensified Maillard reaction. According to Yoo et al. (2020), searing leads to a higher concentration of Maillard reaction products and a decrease in reducing sugars. The higher surface temperature increases due to shearing also contributed to the production of Trp-P-2 at the 200 °C air frying temperature. In a previous study in which beef patties were cooked to 160 and 220 °C, respectively, Trp-P-2 was only detected at the higher cooking temperature of 220 °C (Jamali et al., 2016). This suggests that searing promotes chemical pathways conducive to HCAs formation in beef steak during air frying.

### Effects of air frying conditions on the formation of PAHs in whole chicken and beef steak

PAHs were analyzed in whole chicken and beef steak, with the results were shown in Tables 1 and 2. Among the 4 target PAHs (B[a]P, B[a]A, B[b]F, and CRY), only B[a]P was detected, and only in whole chicken samples. No PAHs were detected in any of the beef steak samples, regardless of cooking condition or searing treatment.

Air frying increased B[a]P levels in chicken compared to raw samples. While raw chicken contained 0.028 µg/kg of B[a]P, air-fried samples showed B[a]P concentrations ranging from 0.032 to 1.141 µg/kg. However, there were no statistically significant differences in B[a]P content among different air frying conditions (*p* > 0.05). Importantly, the maximum B[a]P concentration detected remained below the regulatory limit of 2.0 µg/kg established for meat products by the European Commission (European Commission, 2011).

In contrast, PAHs were not detected in raw or air-fried beef steak in this study. Aaslyng et al. (2013) previously reported PAHs level ranging from 0.4 to 65 µg/kg in beef cooked to core temperatures of 40–80 °C. The absence of PAHs in the current beef steak samples may be attributed to the lower internal temperatures achieved during air frying, which were likely insufficient for PAHs formation. According to Borela et al. (2022), air frying requires a longer cooking time than pan frying to reach the same level of doneness, as measured by internal temperature. The difference in PAHs presence between chicken and beef may also be related to variations in sugar content. According to Tengilimoglu-Metin et al. (2017), raw chicken contains higher amounts of reducing sugars—glucose (0.15 mg/g) and fructose (0.16 µg/kg)—compared to raw beef, which contains only glucose (0.13 mg/g). Since reducing sugars are known precursors for PAHs formation, the higher sugar content in chicken could partially explain the formation of B[a]P in air-fried chicken but not in beef steak.

### Effects of different air frying conditions on acrylamide formation in whole chicken and beef steak

The acrylamide contents in whole chicken and beef steak cooked using an air fryer are presented in Tables 1 and 2. In whole chicken, different air frying conditions did not significantly affect acrylamide formation (*p* > 0.05). The acrylamide levels observed were generally within the range reported in previous studies, which noted concentrations up to 3.49 μg/kg in air-fried chicken breasts, thighs, and wings (Lee et al., 2020).

Overall, acrylamide levels were higher in chicken samples than in beef steak, except when beef was subjected to searing treatment prior to air frying. This difference may be attributed to the higher levels of reducing sugars in raw chicken compared to raw beef. As reported by Tengilimoglu-Metin et al. (2017), chicken contains more reducing sugars such as glucose and fructose, which are key precursors in the Maillard reaction leading to acrylamide formation.

To evaluate the influence of searing, beef steak was seared before air frying. The acrylamide content significantly increased in seared samples compared to non-seared ones. For instance, at 180 °C, seared beef steak contained 2.41 μg/kg of acrylamide, which was approximately 6.2 times higher than the 0.39 μg/kg found in non-seared samples (*p* < 0.05). A similar trend was observed at 200 °C, where seared beef steak exhibited 2.13 μg/kg of acrylamide, compared to 0.98 μg/kg without searing (*p* < 0.05). Yoo et al. (2020) reported that seared beef had lower levels of reducing sugars and higher levels of Maillard reaction products compared to oven-cooked beef, indicating that the Maillard reaction was more actively promoted during searing. This suggests that searing may enhance acrylamide formation possibly by accelerating surface dehydration and concentrating acrylamide precursors, such as free asparagine and reducing sugars. Although this study did not directly compare air frying with other cooking methods, previous studies have reported that traditional frying techniques, such as pan-frying or deep-frying, transfer heat more rapidly than air frying (Teruel et al., 2015), resulting in higher surface temperatures and faster surface dehydration. Lee et al. (2020) also reported that the acrylamide content of deep-fried chicken was higher than that of air-fried chicken. Such differences in heat transfer characteristics may partly explain why acrylamide formation varies among cooking techniques.

### Effect of marination on the formation of HCAs, PAHs, and acrylamide in air-fried whole chicken

The contents of HCAs, PAHs, and acrylamide in unmarinated and marinated air-fried whole chicken are presented in Table 3. Marination with milk and beer significantly reduced the total content of 8 HCAs (*p* < 0.05). Under air frying at 200 °C for 40 min, the total 8 HCAs level were reduced from 2.56 μg/kg to 1.01 μg/kg (60.6% inhibition) with milk and to 1.28 μg/kg (50.0% inhibition) with beer. Among the individual HCAs, the PhIP content was significantly decreased by both milk and beer marinades (*p* < 0.05), with levels reduced to approximately 60.6–70.8% of that in unmarinated samples. However, the effects of marination on AαC and MeAαC formation were less pronounced. These findings align with previous reports that marination can effectively reduce HCAs formation. For example, Knize et al. (2002) showed that marinating chicken before cooking significantly reduced PhIP levels. Similarly, Melo et al. (2008) reported that marinating beef steak with beer or wine for 6 h reduced MeIQx and PhIP levels by about 88% and 40%, respectively.

**Table 3:** Effect of marination on the production of 10 HCAs, 4 PAHs, and acrylamide (μg/kg) and inhibition rates (%) in whole chicken using an air fryer

Sample	Air fryer cooking method	AαC	MeAαC	IQ	MeIlQx	MeIQ	PhIP	Trp-P-1	Trp-P-2	Harman	Norharman	Total 8 HCAs*	Total PAHs	Acrylamide	Inhibitory efficiency of HCAs	Inhibitory efficiency of PAHs	Inhibitory efficiency of acrylamide
Whole chicken (200 °C, 40 min)	Control	0.17 ± 0.03^a^	0.15 ± 0.01^a^	N.D	0.08 ± 0.01^b^	N.D	2.16 ± 0.28^a^	N.D	N.D	0.72 ± 0.02^b^	1.62 ± 0.23^b^	2.56 ± 0.30^a^	0.100 ± 0.10^a^	1.47 ± 1.52^a^	-	-	-
	Marinated in milk	0.10 ± 0.02^b^	0.15 ± 0.01^a^	N.D	0.13 ± 0.01^a^	N.D	0.63 ± 0.08^b^	N.D	N.D	0.55 ± 0.02^b^	2.08 ± 0.28^b^	1.01 ± 0.04^b^	0.035 ± 0.06^a^	2.58 ± 0.80^a^	60.55%	65.00%	-75.51%
	Marinated in beer	0.19 ± 0.02^a^	0.14 ± 0.01^a^	N.D	0.11 ± 0.02^a^	N.D	0.85 ± 0.11^b^	N.D	N.D	1.66 ± 0.27^a^	3.40 ± 0.43^a^	1.28 ± 0.12^b^	0.040 ± 0.07^a^	1.35 ± 0.73^a^	50.00%	60.00%	8.16%

*8 HCAs: AαC, MeAαC, IQ, MeIQx, MeIQ, PhIP, Trp-P-1, and Trp-P-2

N.D. indicates not detected. Data are expressed as mean ± standard deviation

Mean values followed by different lowercase letters within each column for different marination are significantly different for whole chicken (*p* < 0.05)

It has been suggested that the carbohydrate content in milk and beer may contribute to HCAs reduction by enhancing water retention, thus lowering cooking temperature and limiting Maillard reaction progress (Jägerstad et al., 1998). Nonetheless, the effect of milk on MeIQx formation has not been fully investigated. Interestingly, some antioxidants have been reported to reduce MeIQx formation, while others may promote it depending on their composition (Busquets et al., 2006).

As for polycyclic aromatic hydrocarbons (PAHs), their levels also decreased after marination. The total PAHs content declined from 0.100 μg/kg in unmarinated samples to 0.035–0.040 μg/kg after milk or beer marination (Table 3). Among the 4 PAHs (B[a]A, B[b]F, B[a]P, and CRY), only B[a]P was detected. Its content decreased from 0.100 μg/kg to 0.035 μg/kg (milk) and 0.040 μg/kg (beer) after air frying at 200 °C for 40 min. However, the differences in PAHs content between milk- and beer-marinated groups were not statistically significant (*p* > 0.05).

Previous studies have shown that marination with antioxidant-rich extracts can reduce PAHs formation. For example, Shen et al. (2022) found no significant PAHs reduction in duck skin marinated with rosemary extract, whereas significant reductions were observed with green tea, bamboo leaf, and grape seed extracts. Furthermore, PAHs inhibition was dependent on the concentration of the marinating agent, with higher concentrations showing greater effect.

Regarding acrylamide, the acrylamide level in the control (raw chicken) was 1.47 μg/kg. Beer marination resulted in a slight decrease (1.35 μg/kg; 8.16% inhibition), whereas milk marination led to a notable increase (2.58 μg/kg; 75.51% increase). This rise may be due to the lactose and lysine in milk, which are potential precursors of the Maillard reaction. Additionally, unlike beer, which contains polyphenols and antioxidants that may suppress acrylamide formation (Piazzon et al., 2010), milk contains relatively low levels of such inhibitory compounds. Therefore, the lack of antioxidant protection in milk-marinated samples might have allowed acrylamide precursors to participate more freely in the reaction pathway, resulting in higher acrylamide yields. However, these differences were not statistically significant (*p* > 0.05).

It should be noted that different chicken cuts (e.g., breast, thigh, wing) vary in fat, water, and protein contents, which may influence the formation of HCAs, PAHs, and acrylamide. In this study, edible parts were homogenized to create composite samples in order to minimize variability among replicates and to provide an integrated estimate of consumer exposure. While this approach reflects realistic whole-chicken consumption, it does not allow assessment of the contribution of individual cuts. Future studies that compare toxicant formation across specific parts in relation to their compositional differences would therefore be valuable.

### Effect of spices on the production of HCAs, PAHs, and acrylamide in air-fried beef steak

The contents of HCAs in air-fried beef steak cooked with different spices are presented in Table 4. The addition of rosemary, turmeric, and garlic significantly affected HCAs levels (*p* < 0.05). All three spices markedly reduced the formation of HCAs, including AαC, MeAαC, Trp-P-2, and PhIP. Compared to the control, the PhIP content was significantly reduced by 64.4–69.4% in spiced samples (*p* < 0.05).

**Table 4:** Effect of spices on the formation of 10 HCAs, 4 PAHs, and acrylamide (μg/kg) and inhibition rates (%) in air-fried beef steak

Sample	Air fryer cooking method	AαC	MeAαC	IQ	MelIQx	MeIQ	PhIP	Trp-P-1	Trp-P-2	Harman	Norharman	Total 8 HCAs*	Total PAHs	Acrylamide	Inhibitory efficiency of HCAs	Inhibitory efficiency of PAHs	Inhibitory efficiency of acrylamide
Beef steak (Searing, 200 °C, 13 min)	Control	0.09 ± 0.02^a^	0.10 ± 0.00^a^	N.D	N.D	N.D	1.92 ± 0.31^a^	N.D	0.09 ± 0.01^a^	1.01 ± 0.17^b^	4.17 ± 0.04^ab^	2.19 ± 0.32^a^	N.D	2.13 ± 0.15^b^	-	-	-
	Rosemary	0.07 ± 0.01^a^	0.08 ± 0.00^b^	N.D	N.D	N.D	0.58 ± 0.11^b^	N.D	N.D	0.65 ± 0.02^c^	4.14 ± 0.03^b^	0.73 ± 0.12^b^	N.D	1.65 ± 0.10^c^	66.67%	-	22.54%
	Turmeric	N.D	0.08 ± 0.00^b^	N.D	N.D	N.D	0.59 ± 0.09^b^	N.D	N.D	0.37 ± 0.01^c^	4.19 ± 0.01^a^	0.67 ± 0.09^b^	N.D	0.92 ± 0.19^d^	69.41%	-	56.81%
	Garlic	0.07 ± 0.02^a^	0.09 ± 0.01^b^	N.D	N.D	N.D	0.63 ± 0.05^b^	N.D	N.D	1.40 ± 0.25^a^	4.17 ± 0.01^ab^	0.78 ± 0.07^b^	N.D	5.66 ± 0.29^a^	64.38%	-	-165.7%

*8 HCAs: AαC, MeAαC, IQ, MeIQx, MeIQ, PhIP, Trp-P-1, and Trp-P-2

N.D. indicates not detected. Data are expressed as mean ± standard deviation

Mean values followed by different lowercase letters within each column for different spices are significantly different for beef steak (*p* < 0.05)

Among the spices, turmeric showed the greatest inhibitory effect on total 8 HCAs (69.4%), followed by rosemary (66.7%) and garlic (64.4%). The reduction in HCA formation is likely associated with the total phenolic content (TPC) of the spices, which contributes to their radical scavenging and antioxidant activity, as reported in previous studies (Puangsombat et al., 2011). For instance, turmeric reduced HCAs by 39%, and this effect correlated positively with TPC and antioxidant capacity. Similarly, MeIQx and PhIP levels in beef steak were significantly reduced by the addition of rosemary (43.5%) and turmeric (39.2%) prior to frying (Puangsombat et al., 2011). In another study, the inclusion of garlic and onion in fried meatballs and beef decreased IQ and PhIP concentrations (Lu et al., 2018).

Table 4 also shows the effects of the spices on the levels of four PAHs and acrylamide in air-fried beef steak. None of the four PAHs were detected in beef steak samples, regardless of spice addition. However, in a previous study, garlic significantly reduced PAHs level by 65.1% in beef meatballs (Lu et al., 2018). The absence of detectable PAHs in this study may be attributed to the relatively lower cooking temperatures and enclosed heating mechanism of air frying, which minimizes fat pyrolysis and smoke production which are primary sources of PAHs in grilled or pan-fried meat.

Spices had a significant impact on acrylamide formation (*p* < 0.05). Among the tested spices, rosemary and turmeric significantly reduced acrylamide levels, with inhibition rates of 22.54% and 56.81%, respectively, compared to the control. This inhibitory effect is likely attributable to their high antioxidant capacities, particularly due to the presence of phenolic compounds such as carnosol, rosmanol, and rosmaquinone in rosemary.

Conversely, garlic markedly increased acrylamide formation, with levels reaching 5.66 μg/kg. This increase may be due to the relatively high content of reducing sugars in garlic, which can serve as precursors in the Maillard reaction. Previous studies have reported that garlic contains 3.34–3.97 g/100 g of reducing sugars (Kang et al., 2020), which is substantially higher than the trace amounts typically found in rosemary and turmeric. In addition, Sipahi et al. (2024) reported that garlic extract enhanced acrylamide formation in French fries, attributing this effect to the presence of chlorogenic acid. The TPC and antioxidant capacities of turmeric, rosemary, and garlic have been evaluated previously, with rosemary showing the highest TPC and antioxidant activity (Kwon et al., 2023). Turmeric contains curcuminoids such as curcumin, bisdemethoxycurcumin, and demethoxycurcumin, and has been found to be as effective as rosemary in reducing HCA formation (Cao et al., 2020). Curcumin can react with the α-amino group of asparagine during heating to form a Schiff base, potentially influencing acrylamide formation (Hamzalıoğlu et al., 2013).

### Correlations between HCAs, PAHs, and acrylamide contents in whole chicken and beef steak

**Fig. 2:**
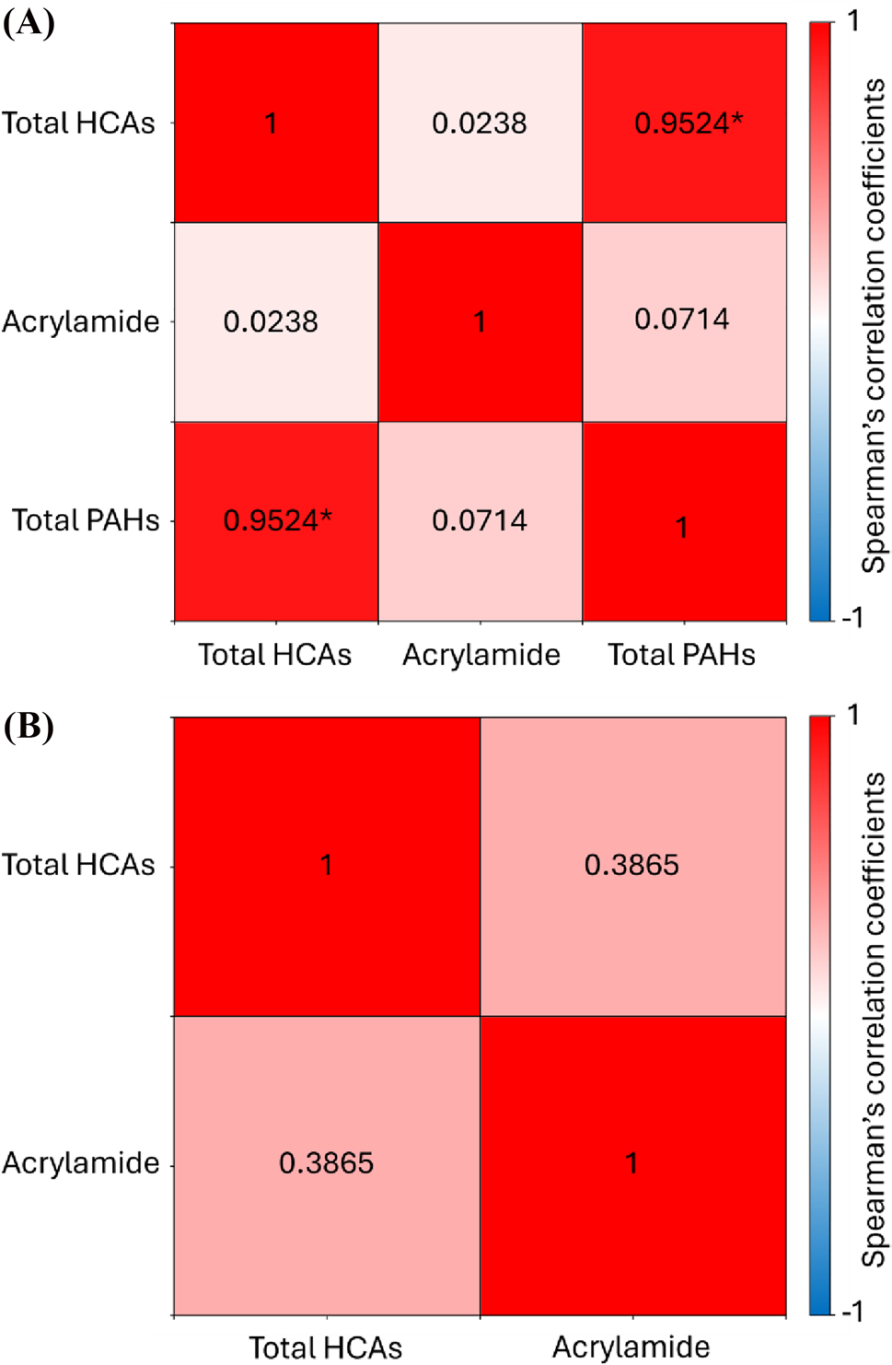
Spearman’s correlation coefficients between the levels of total HCAs, PAHs, and acrylamide in (**A**) whole chicken and (**B**) beef steak. *p < 0.05 were considered as statistically significant

The Spearman correlation coefficients between HCAs, PAHs, and acrylamide are presented in Fig. 2. In whole chicken, a significant positive correlation was observed between total HCA and PAH levels (*p* < 0.01), suggesting a shared thermal formation mechanism. In contrast, no significant correlation was found between acrylamide and either HCAs or PAHs (*p* > 0.01). A similar trend was observed in beef steak, where PAHs were not detected under any cooking condition, and no correlation was found between HCAs and acrylamide (*p* > 0.01).

Although HCAs, PAHs, and acrylamide can all form via Maillard-type reactions during cooking, their precursor specificity and formation conditions differ substantially. HCAs are primarily derived from reactions among creatinine, amino acids, and sugars at elevated surface temperatures, while PAHs are produced mainly via the pyrolysis of organic matter, such as fat and protein, under open flame or smoke-rich environments. In contrast, acrylamide formation predominantly involves the reaction of asparagine with reducing sugars and occurs at relatively lower temperatures. These differences in chemical pathways and thermal dependencies likely explain the observed lack of correlation between acrylamide and the other two compound classes in both meat types.

Overall, these findings suggest that acrylamide is generated via a mechanistically distinct route compared to HCAs and PAHs and may be less influenced by the thermal intensity or combustion-related factors common to high-temperature meat cooking. Further studies are warranted to elucidate the interplay among these toxicants under varied cooking systems, ingredients, and food matrices.

To summarize, air frying conditions, including temperature, cooking time, searing marination, substantially influenced the formation of HCAs, PAHs, and acrylamide in whole chicken and beef steak. Elevated air frying temperatures and searing promoted the formation of HCAs and acrylamide, particularly in beef steak. In contrast, marinating whole chicken with milk or beer effectively reduced HCA levels without significantly affecting PAHs or acrylamide. The addition of spices such as turmeric, rosemary, and garlic significantly suppressed HCA formation in beef, with turmeric showing the strongest inhibitory effect. However, garlic increased acrylamide levels, likely due to its high reducing sugar and chlorogenic acid content. These findings, in combination with the observed correlations, indicate that HCAs and PAHs share a similar thermal formation mechanism, whereas acrylamide follows a distinct chemical pathway. The results highlight the importance of selecting appropriate ingredient treatments and cooking conditions, particularly the use of turmeric in beef and marination in chicken, to mitigate the formation of hazardous compounds during air frying.

## Supplementary Information

Below is the link to the electronic supplementary material.

Supplementary file1 (DOC 79 KB)
