# Supplementary material for: Mitigation of heterocyclic amines, polycyclic aromatic hydrocarbons, and acrylamide in air-fried chicken and beef: effects of cooking methods and marinades
Source: Food Sci Biotechnol. 2025 Sep 27;34(16):3873–85. doi: 10.1007/s10068-025-02005-8 (PMC12589718; doi:10.1007/s10068-025-02005-8)
Supplement: Supplementary file 1 — Supplementary file1 (DOC 79 KB) [file 10068_2025_2005_MOESM1_ESM.doc]

**Table** **S1.** LC-MS/MS acquisition parameters used for the analysis of 10 HCAs and 8 HCAs isotopes.

| Compound | Precursor ion (*m/z*) | Product ions (*m/z*) | Fragment voltage (V) | Collision energy (eV) |
| --- | --- | --- | --- | --- |
| IQ | 199 | **184**, 157 | 173 | **30**, 36 |
| MelQ | 213 | **198**, 145 | 165 | **30**, 30 |
| MelQx | 214 | **199**, 173 | 159 | **30**, 26 |
| PhIP | 225 | **210**, 183 | 181 | **32**, 36 |
| AαC | 184 | **167**, 157 | 153 | **24**, 24 |
| MeAαC | 198 | **181**, 129 | 153 | **24**, 30 |
| Trp-P-1 | 212 | **195**, 168 | 161 | **26**, 32 |
| Trp-P-2 | 198 | **181**, 154 | 151 | **26**, 32 |
| Harman | 183 | **115**, 168 | 159 | **34**, 32 |
| Norharman | 169 | **115**, 89 | 191 | **32**, 36 |
| IQ-*d*_3_ | 202 | **184**, 131 | 159 | **30**, 32 |
| MeIQ-*d*_3_ | 216 | **198**, 145 | 161 | **30**, 32 |
| MeIQx-*d*_3_ | 217 | **199**, 173 | 163 | **30**, 26 |
| PhIP-*d*_3_ | 228 | **210**, 157 | 179 | **34**, 32 |
| MeAαC-*d*_3_ | 201 | **184**, 132 | 155 | **24**, 30 |
| Trp-P-2-^13^C_2_-^15^N | 201 | **155**, 183 | 163 | **34**, 24 |
| Harman-*d*_3_ | 186 | **115**, 168 | 161 | **34**, 32 |
| Norharman-*d*_7_ | 176 | **120**, 148 | 169 | **34**, 32 |

Heterocyclic amines (HCAs) were quantified in MRM mode. Product ion and collision energy in bold and not in bold were used for quantifier ion transitions and qualifier ion transitions, respectively.
